# Stratified Fitness: Post-COVID Inequities in Physical Education Across Race, Class, and Dis/Ability in Connecticut Schools

**DOI:** 10.3390/children12091144

**Published:** 2025-08-28

**Authors:** Da’Shay Templeton, Ruslan Korchagin

**Affiliations:** Educational Leadership Department, Graduate School of Education, California Lutheran University, 60 W. Olsen Road, Thousand Oaks, CA 91360, USA; rkorchag@callutheran.edu

**Keywords:** physical fitness, educational equity, COVID-19 pandemic, stratification economics, culturally responsive education, physical education

## Abstract

**Background/Objectives:** This study investigates disparities in physical fitness among Connecticut K-12 students post-COVID-19, highlighting persistent inequities across race, socioeconomic status, language proficiency, and dis/ability. **Methods**: Utilizing state-wide data from Connecticut’s EdSight portal, the research reveals a significant decline in student physical fitness compared to pre-pandemic levels, notably lagging behind academic recovery. **Results**: Findings underscore stark disparities, with marginalized groups such as students with disabilities, English learners, and racially minoritized students consistently performing lower than their peers. District-level analyses further illustrate pronounced inequities, where affluent, predominantly White districts achieved nearly four times higher fitness scores compared to economically disadvantaged, racially diverse districts. **Conclusions**: These disparities reflect systemic resource allocation issues and align with the framework of stratification economics, emphasizing structural inequities rather than individual deficits. The study advocates for equity-based funding, mandatory physical education monitoring, culturally responsive teaching practices, and systemic policy reforms to ensure equitable access to physical education resources and holistic student well-being.

## 1. Introduction

Physical education often plays a secondary role in the educational hierarchy, despite its vital importance in supporting students’ long-term physical, mental, and cognitive well-being [1]. For students from historically underserved backgrounds—such as racially minoritized youth, English learners, students with disabilities, and those from low-income families—school-based physical education may be their only consistent opportunity for structured physical activity. When access to quality physical education is limited, the resulting health disparities are not isolated; they intersect with academic challenges, reduced self-esteem, and broader social exclusion [2]. Physical education, therefore, is not a luxury or an optional extracurricular activity—it is a civil rights issue and a crucial aspect of educational equity.

The COVID-19 pandemic intensified existing disparities. As schools shifted to remote and hybrid instruction, physical education was among the first subjects to be reduced or suspended [3]. Even after a return to in-person learning, many schools continued to allocate fewer resources, instructional minutes, and accountability mechanisms to PE relative to core academic subjects [4]. This uneven recovery has disproportionately impacted under-resourced districts, where students already contend with barriers such as community disinvestment, inadequate facilities, and overcrowded schools [3]. In this context, examining post-pandemic trends in student physical fitness becomes critical for identifying persistent structural inequities embedded in the education system.

The pandemic and its aftermath highlighted and worsened existing inequalities in health, education, and economic stability. Public health measures like lockdowns and stay-at-home orders disrupted daily life, limiting access to physical activity and increasing social isolation. This was especially true in low-income and urban areas, where families also faced job losses, food insecurity, mental health struggles, and caregiving challenges [5]. These burdens disproportionately affected racially minoritized and economically disadvantaged groups. Even after the pandemic, many vulnerable populations continued to face barriers to recovery, such as limited healthcare, underfunded schools, and fewer opportunities for physical activity. These challenges affected not only student well-being but also schools’ and families’ ability to support physical education effectively.

Physical activity is one of the most effective ways to promote mental and physical health, prevent diseases, and strengthen the immune system [6]. It also enhances cognitive functions such as attention, memory, inhibition, and executive functioning, which are linked to improved academic performance [7]. A meta-analysis of moderate-to-vigorous physical activity in children and adolescents confirmed its moderate positive impact on academic performance [8]. Despite these benefits, physical activity is often undervalued in schools [9]. A recent study of physical education in schools in southern California highlighted this issue, showing that physical education has been significantly undervalued compared to other academic subjects. Following COVID-19 disruptions, many schools reduced physical education instruction, reallocated budgets to core subjects, and further diminished its importance through weak accountability and unclear fitness assessments [4].

Building on this concern, research shows that the lack of attention to physical education disproportionately affects the most vulnerable and minoritized student groups. Historically underserved populations, including ethnic minorities, low-income individuals, and those with physical disabilities, are less likely to engage in sufficient moderate-to-vigorous physical activity, increasing their risk of mortality and morbidity [10]. Socioeconomic disparities exacerbate these issues, as students from lower socioeconomic backgrounds tend to participate less in physical activity compared to their peers [4]. Racially minoritized youth, especially those from non-English-speaking households, face additional barriers such as fear of injury, social exclusion, high costs, and transportation difficulties, leading to lower participation rates in sports and physical activity [11]. Moreover, students with disabilities often experience bullying, social isolation, and discrimination in physical education classes [12,13].

Systemic resource gaps across communities further exacerbate these disparities; as a result, minoritized students consistently demonstrate lower levels of physical fitness than their more affluent peers, with racial and socio-economic inequalities being particularly prominent [14]. Wealthier families are more likely to provide their children with access to high-quality education, including private tutoring and extracurricular activities, such as sports lessons [15]. Predominantly White, wealthier districts benefit from greater economic advantages, leading to better facilities, more experienced teachers, and a broader range of educational opportunities [16]. In sports, the financial gap is evident: families earning less than $50,000 spend an average of $523 on their children’s primary sport, while families earning $50,000 to $149,999 pay $940, and those earning over $150,000 spend approximately $2068. Low-income children are more likely to participate in lower-cost sports if they can afford to do so in the first place [17]. To change the situation, these students urgently need legislation to support their physical and academic development, which in some states, such as California, is provided through targeted funding. The Local Control Funding Formula in California allocates resources based on student needs, offering additional funding to disadvantaged communities, including racially minoritized students, English language learners, foster youth, homeless youth, and students eligible for free or reduced lunch [18]. The idea of providing assistance to minoritized students rather than stigmatizing them aligns with the assumptions of stratification economics and the concept of culturally responsive teaching, which proposes that educators reduce deficit thinking and instead focus on providing a high-quality education, equipping students with the necessary resources for success, and recognizing and leveraging the unique strengths of these students [19].

This study also applies the theory of stratification economics to examine racial, economic, and dis/ability-based disparities in physical education and academic performance in Connecticut following the COVID-19 pandemic. Stratification cconomics, developed as a response to the shortcomings of neoclassical economic theories, emphasizes structural and institutional mechanisms that sustain intergroup hierarchies and perpetuate inequality across generations [20,21].

Unlike approaches that attribute disparities to individual shortcomings, stratification economics argues that these differences arise from systemic resource transfers, group-based marginalization, and institutional discrimination. According to this framework, dominant social groups—defined by race, class, or other characteristics—actively preserve their privilege by controlling access to education, extracurricular activities, nutrition, and public health resources [15,22]. These inequalities are deeply rooted in historical systems of advantage.

By applying the principles of stratification economics, this study reveals that physical fitness performance in schools is shaped not by individual choice but by structural privilege and deprivation. The data highlight a disproportionate decline in physical fitness performance among racially minoritized students, English language learners, and students with disabilities, underscoring unequal investments in health and education based on socioeconomic status.

Choosing Connecticut for this study was not a random decision, but rather a deliberate one, as Connecticut is one of the few U.S. states that systematically tracks and publicly reports detailed information regarding students’ physical fitness performance. Located in the northeastern United States, Connecticut has a diverse student population and a strong tradition of statewide educational data collection, making it a valuable context for studying post-pandemic disparities. Although the primary focus of this study is on disparities in physical fitness performance among Connecticut students, academic performance data is included as a benchmark to provide context for the extent of post-pandemic recovery across different educational areas. This comparison helps determine whether the recovery in physical fitness has matched the pace of academic recovery, underscoring how systemic neglect of physical education disproportionately impacts high-needs student groups.

While the benefits of physical activity are widely acknowledged, disparities in access, participation, and institutional support continue to hinder equitable health and educational outcomes for vulnerable student populations. These inequities stem not just from individual factors, but from systemic decisions regarding funding, priorities, and accountability within the education system. As schools address the academic and health impacts of the COVID-19 pandemic, it is essential to explore how these disparities affect both physical fitness and academic performance. This study aims to contribute to this area by examining post-pandemic trends in Connecticut schools, with a focus on differences across socioeconomic, racial, linguistic, and dis/ability-based groups.

## 2. Materials and Methods

### 2.1. Research Design

This study utilizes a quantitative, descriptive–comparative design to explore differences in student physical fitness and academic performance in Connecticut before and after the COVID-19 pandemic. It analyzed publicly available administrative data to identify patterns of inequity across demographic and district-level factors. The findings were interpreted through the lens of stratification economics, emphasizing systemic inequities over individual-level explanations.

For the purposes of this study, the term “Black Americans” refers to students identified in state data as Black or African American. The term “Latin”* refers to students identified as Hispanic or Latino in state reporting.

### 2.2. Participants

The study utilizes publicly available statewide administrative data from the Connecticut State Department of Education’s EdSight portal to explore the effects of the COVID-19 pandemic on students’ physical fitness performance [23]. It focuses on public school students in Grades 4, 6, 8, and high school across districts that participated in the Connecticut Physical Fitness Assessment during the 2018–2019, 2021–2022, 2022–2023, and 2023–2024 academic years.

In total, the analysis covers data from over 160 school districts and hundreds of public schools statewide. During the 2023–2024 academic year, more than 100,000 students participated in the Connecticut Physical Fitness Assessment, reflecting a diverse population in terms of race, ethnicity, socioeconomic status, language background, and dis/ability.

### 2.3. Data Sources and Procedures

The data for this study were chosen based on three key criteria: (1) accessibility through the Connecticut State Department of Education’s EdSight portal; (2) relevance to the relationship between physical fitness and academic performance for K–12 public school students; and (3) completeness and consistency across the academic years analyzed. To ensure robust and meaningful comparisons, only districts that administered the Connecticut Physical Fitness Assessment and had at least 100 enrolled students in the 2023–2024 academic year were included in the analysis of districts with the highest and lowest physical fitness scores.

The Connecticut Physical Fitness Assessment identifies the “Health-Related Fitness Zone” as scores linked to good health; students meeting or exceeding this benchmark are classified as achieving the Healthy Fitness Zone. The terms “Reaching Healthy Fitness Zone”, “Physical Fitness Test Assessment,” and “Physical Fitness Performance” are used interchangeably in this article. The physical fitness performance of students before and after the COVID-19 pandemic is also compared with their academic performance in English Language Arts (ELA), Mathematics, and Science [23]. The study also examined disparities across student profiles and district demographics in Connecticut.

Academic performance data, including ELA, math, and science scores for the academic years 2018–2019 (pre-pandemic baseline), 2021–2022, 2022–2023, and 2023–2024 were obtained from EdSight, using Connecticut’s Performance Index (scaled 0–100) with a state target of 75 for all student groups. Science performance data from 2018–2019, aligned with the Next Generation Science Standards, serve as a baseline and are not directly comparable to earlier years.

To examine performance differences across student subgroups, we extracted data for the 2023–2024 academic year disaggregated by race/ethnicity, gender, English Learner (EL) status, disability status, and high-needs status. In Connecticut’s reporting framework, high-needs students are defined as those who belong to at least one of the following categories: economically disadvantaged (eligible for free/reduced-price meals), English Language Learners, or students with disabilities [23]. The top and bottom five districts in physical fitness performance were identified, and demographic profiles were compiled for these districts, focusing on the aforementioned subgroups.

### 2.4. Data Analysis

The analysis employed a descriptive and comparative approach to examine trends in physical fitness and academic performance over time, both before and after the pandemic. Subgroup comparisons were made based on race, English Learner status, dis/ability, gender, and high-needs designation. Additionally, district-level analyses compared the top and bottom five districts in physical fitness performance for the 2023–2024 academic year, with median demographic characteristics calculated to identify patterns linked to student outcomes.

### 2.5. Data Validation

The data for this study were obtained from the Connecticut State Department of Education’s EdSight portal, which provides standardized and audited information at the state, district, and school levels. These data are reliable due to the state’s standardized reporting processes and quality control measures. To ensure accuracy, the datasets were checked for completeness across all variables and cross-referenced with state technical documentation.

## 3. Results

An analysis of Connecticut state-level data from 2018–2019 to 2023–2024 highlights significant trends in student performance across academic subjects and physical fitness. Before the COVID-19 pandemic, during the 2018–2019 school year, 67.7% of students met or exceeded the state performance index target in English Language Arts (ELA), followed by 63.8% in Science, 63.1% in Math, and 52.9% achieving the Healthy Fitness Zone in physical fitness assessments. These results indicate that even before the pandemic, students consistently underperformed in physical fitness compared to other academic subjects.

**Figure 1:**
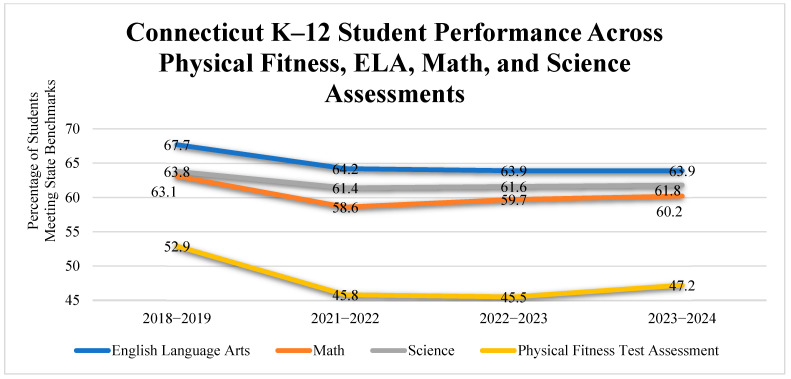
Connecticut K–12 student performance across Physical Fitness, ELA, Math, and Science assessments.

The pandemic’s disruption caused declines across all domains by 2021–2022. Physical fitness saw the steepest drop, with only 45.8% of students meeting the benchmark—a decrease of over seven percentage points. While modest improvements occurred in later years, physical fitness performance remained below pre-pandemic levels, reaching just 47.2% in 2023–2024. Academic subjects showed a more stable recovery: ELA performance rebounded to 63.9%, Math improved to 60.2%, and Science rose slightly to 61.8% by the 2023–2024 school year. These trends suggest a slower and uneven recovery in physical fitness compared to academic performance. See Figure 1 for detailed information regarding Connecticut K-12 student performance across physical fitness, ELA, math, and science assessments.

**Figure 2:**
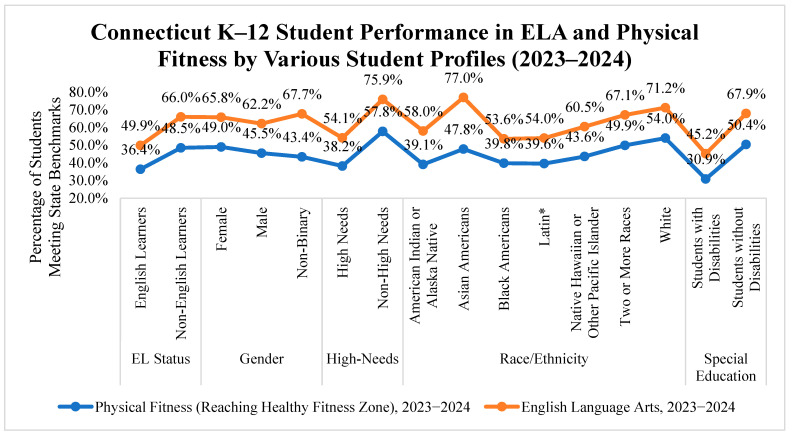
Connecticut K–12 student performance in ELA and Physical Fitness by various student profiles (2023–2024).

To investigate disparities in post-pandemic recovery, performance in physical fitness and English Language Arts (ELA)—the highest-scoring academic subject—was compared across demographic groups for the 2023–2024 academic year. In every subgroup analyzed, ELA performance consistently outpaced Physical Fitness, often by significant margins. Vulnerable groups, including students with disabilities, English Language Learners, high-needs students, American Indian or Alaska Native, Black Americans, and Latin* students, showed the lowest physical fitness scores among all other groups. Detailed comparisons of ELA and Physical Fitness performance by various student groups are available in Figure 2.

Students with disabilities had the lowest physical fitness scores, with only 30.9% meeting the benchmark, compared to 45.2% in ELA. Non-disabled peers performed better, with 50.4% reaching the fitness zone and 67.9% achieving the ELA target. English Language Learners also lagged, with 36.4% meeting the fitness standard versus 49.9% in ELA—a 13.5 percentage point gap. Non-English Language Learners showed a wider gap of 17.5 percentage points (48.5% in fitness vs. 66.0% in ELA). High-needs students consistently underperformed compared to their non-high-needs peers. Only 38.2% of high-needs students met the physical fitness standard, while 54.1% achieved the ELA benchmark. Non-high-needs students performed significantly better, with 57.8% meeting fitness standards and 75.9% meeting ELA targets.

Racial and ethnic disparities were also evident. White American students had the highest fitness score (54.0%) and demonstrated strong English Language Arts (ELA) performance (71.2%). In contrast, Latin* and Black American students had notably lower scores in both domains, with physical fitness rates below 40%. American Indian or Alaska Natives had the lowest physical fitness score of 39.1 among all other racial groups. Asian American students showed the most significant gap between physical fitness (47.8%) and ELA (77.0%), a difference of 29.2 percentage points.

These findings emphasize the need for targeted interventions to improve physical fitness outcomes, particularly for vulnerable student groups such as students with disabilities, English Language Learners, high-needs students, American Indian or Alaska Native, Black Americans, and Latin* students. Addressing these disparities may require policy and financial actions to ensure equitable access to resources and support.

To explore the structural and contextual factors influencing physical fitness outcomes, five Connecticut school districts with the highest and lowest fitness performance in the 2023–2024 academic year were analyzed. Refer to Table 1 and Table 2 to see Connecticut school districts with the lowest and the highest physical fitness assessment scores.

The lowest-performing districts—Jumoke Academy District, Goodwin University Educational Services, Thompson, Torrington, and Achievement First Bridgeport Academy—had a median fitness score of just 20.3%. These districts had high proportions of high-needs students (median: 75.9%), significant populations of English Language Learners (13.5%), and racially diverse student bodies, with Latin* and Black American students comprising 41.2% and 32.6%, respectively. Students with disabilities represented a median of 17.6% in these districts.

In contrast, the highest-performing districts—Capital Preparatory Harbor, Greenwich, New Canaan, Lisbon, and Winchester—had a median fitness score of 78.3%. These districts served fewer English Language Learners (median: 4.8%) and had a predominantly White American student population (77.3%). High-needs students comprised a smaller portion (50.5%), and the rate of students with disabilities was slightly lower (15.4%).

**Figure 3:**
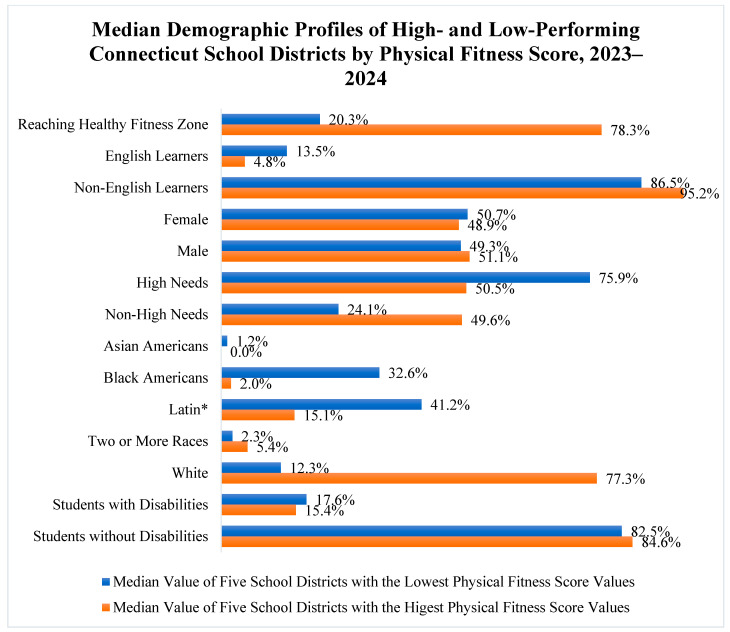
Median demographic profiles of high- and low-performing Connecticut school districts by Physical Fitness score, 2023–2024.

Direct comparisons between the highest- and lowest-performing districts revealed stark disparities. The median fitness score in the highest-performing districts was nearly four times greater than that of the lowest-performing group (78.3% vs. 20.3%). English Language Learner enrollment was almost three times higher in the lowest-performing districts (13.5% vs. 4.8%), and Latin* students were more than twice as prevalent (41.2% vs. 15.1%). Black American students were significantly underrepresented in the highest-performing districts (2.0%) and severely overrepresented in the lowest-performing districts (32.6%). Conversely, White students were significantly underrepresented in the lowest-performing districts (12.3% vs. 77.3%). High-needs students comprised the majority in lower-performing districts (75.9%), whereas they represented only half of the population in higher-performing districts (50.5%). Refer to Figure 3 to see a detailed comparison of Connecticut school districts with the highest and lowest physical fitness assessment scores by various student groups in 2023–2024.

These results underscore the close alignment between demographic composition—particularly race, language status, and economic need—and physical fitness outcomes in Connecticut schools. They raise critical questions about equity in access to physical education and health-supportive resources during the post-COVID recovery period.

## 4. Discussion

This study reveals persistent and substantial disparities in physical fitness performance, as demonstrated by the 2023–2024 state assessment data presented in Section 3, particularly based on race, language status, economic need, and dis/ability. Only 30.9% of students with disabilities met the fitness benchmark, compared to 50.4% of their non-disabled peers. Similarly, English Language Learners scored 36.4%, while non-English Language Learners scored 48.5%. Racial and ethnic disparities were also evident, with Latin* and Black American students scoring below 40%, compared to 54.0% for White American students. The gap between the highest- and lowest-performing districts was especially pronounced, with scores of 78.3% and 20.3%, respectively—a nearly fourfold difference.

This study reveals ongoing and substantial disparities in physical fitness performance among Connecticut K–12 students in the post-COVID era, particularly across racial, linguistic, socioeconomic, and dis/ability categories. These results mirror national trends, indicating that students from historically underserved communities encounter systemic obstacles that hinder equitable access to quality physical education [6,11].

These disparities are not random or a result of individual effort but are rooted in systemic inequities. The findings align with the framework of stratification economics, which suggests that dominant social groups sustain their privilege by limiting resources and marginalizing others. Specifically, students from historically underserved groups, such as racial minorities, English Language Learners, and those with disabilities, show significantly lower physical fitness outcomes due to existing structural barriers rather than personal failings.

Physical activity is well established in the literature as beneficial for both physical and cognitive development, with positive links to academic achievement [7,24]. However, despite these benefits, physical education remains undervalued in many school systems [9,14]. This issue became even more pronounced during and after the COVID-19 pandemic, when schools prioritized academic recovery over physical education and extracurricular activities. As a result, while academic subjects have seen some recovery, physical fitness, especially among vulnerable student groups, has lagged significantly behind. The findings align with those of a previous study, which revealed that physical fitness outcomes fell significantly behind academic indicators as schools prioritized academic subject tests during pandemic recovery efforts [4]. Similarly, another study highlighted how reductions in physical education instructional time, combined with inadequate teacher support and structural inequities, disproportionately affected students in low-income and racially diverse districts [3].

This uneven recovery reflects what stratification economics describes as the compounding effects of intergenerational resource deprivation. Students from high-need backgrounds—many of whom are Black Americans, Latin*, English Language Learners, or have disabilities—were already more likely to have limited access to quality physical education and extracurricular activities before the pandemic, a disparity that continued afterward. Their significantly lower physical fitness outcomes compared to their more affluent or non-minoritized peers highlight the ongoing systemic inequities in education funding, health infrastructure, and community investment. Scholars in the field of stratification economics in education argue that disparities are perpetuated by systemic inequities and the active preservation of privilege by dominant groups [20,21]. The steeper declines in physical fitness among students from racially and economically marginalized backgrounds cannot be attributed to individual effort but are instead the result of structural inequities, such as limited access to extracurricular sports, fewer safe recreational spaces, and underfunded school programs.

Culturally responsive teaching principles emphasize the importance of recognizing the cultural strengths of historically marginalized groups rather than viewing them as deficits [19]. The fact that students from communities with higher proportions of English Language Learners, low-income families, and racially minoritized populations had the lowest fitness scores indicates a lack of inclusive and equitable physical education environments. This finding aligns with previous research, which has shown that these students often face significant logistical and emotional barriers to participation, including limited transportation, social exclusion, and discrimination [11,12].

The gap in physical fitness achievement between the highest- and lowest-performing districts was particularly stark, with wealthier, predominantly White districts achieving nearly four times the fitness levels of more diverse, high-need districts. These disparities are not only a result of inequities in school-based programming but also reflect broader differences in community resources, such as access to parks, sports programs, and safe outdoor spaces. These disparities are often influenced by racialized housing and zoning policies that have historically limited investment in marginalized communities.

To address the current situation, the following policy changes are crucial. First, equitable funding for physical education should be based on students’ needs rather than the location of their neighborhood. Second, mandatory monitoring of physical fitness performance in schools is essential. While Connecticut is one of the few states that tracks students’ physical fitness performance, this practice should be implemented nationwide. The goal is not merely to collect data for reporting purposes but to identify disparities in students’ physical health and provide necessary interventions to help every student succeed both academically and physically. Third, there is a need to adopt targeted funding models, such as the California Local Control Funding Formula, in other states to ensure that marginalized students receive the highest quality education and have access to the resources they need to succeed. Fourth, physical education should be reframed as an essential part of the curriculum that contributes to student success, rather than being viewed as an expendable enrichment activity. Fifth, there is a need to incorporate culturally responsive teaching in physical fitness programs to ensure that every student feels included and affirmed. This change would require physical education teachers to receive appropriate training to avoid cultural exclusion and promote inclusivity.

The findings of this study have significant practical implications for policymakers, educators, and community stakeholders dedicated to promoting equity in education and student well-being. For state and district policymakers, the clear disparities across race, language, and socioeconomic lines underscore the need for equity-based funding models, such as California’s Local Control Funding Formula, to be adapted and implemented in Connecticut and other states. For school and district administrators, the results highlight the urgency of redefining physical education as a core academic subject that supports cognitive, social, and emotional development, rather than treating it as an optional enrichment activity. For community stakeholders and public health organizations, the persistent post-pandemic inequities in physical fitness performance present opportunities to collaborate with schools in expanding access to after-school and community-based physical activity programs, especially in neighborhoods with limited safe recreational spaces. Finally, the study emphasizes the importance of longitudinal data collection and reporting; states without robust, disaggregated fitness monitoring systems should consider adopting Connecticut’s transparent reporting model to better identify inequities and assess the impact of targeted interventions over time.

This study used publicly available, aggregated data from Connecticut’s EdSight portal, which restricted the analysis to descriptive statistics. Without access to individual-level data, inferential statistical methods could not be applied, making it important to interpret the findings within the state-level context. Furthermore, the fitness assessments and academic performance data may not fully reflect broader aspects of student well-being or physical activity outside of school.

Despite its descriptive design, this study makes several important contributions to the literature on educational equity and physical fitness. First, it offers one of the most comprehensive post-COVID analyses of state-level physical fitness outcomes, using publicly available, standardized data from Connecticut—a state that uniquely tracks and reports such information. This detailed approach provides a clearer understanding of how systemic inequities intersect with race, language status, socioeconomic need, and dis/ability to shape fitness outcomes, a perspective that has been largely overlooked in previous research relying on smaller, localized datasets. Second, by applying the lens of stratification economics to statewide fitness and academic data, the study advances theoretical discussions by framing fitness disparities as structurally produced rather than individually driven, building on prior scholarship on educational and health inequities. Finally, the inclusion of district-level comparisons between the highest- and lowest-performing districts provides actionable insights for policymakers, emphasizing the need for equity-focused interventions at both state and local levels.

Future research should aim to access disaggregated, student-level data to enable inferential analysis and more accurate modeling of causal relationships. Longitudinal studies investigating the long-term effects of reduced physical education access during the pandemic would also provide valuable insights. Furthermore, qualitative research exploring the experiences of students and educators—especially in underserved communities—could offer a deeper understanding of the factors contributing to disparities in physical fitness outcomes.

This discussion reinforces the key insight of stratification economics of education: disparities in education and health are not simply the result of individual effort, but stem from systemic resource distribution that favors already privileged groups. In the case of physical fitness, schools have often mirrored and perpetuated broader societal inequities, deprioritizing physical education in ways that disproportionately harm students from racially minoritized, low-income, multilingual, and disabled backgrounds. Without intentional policy changes, these patterns will continue, perpetuating inequality under the guise of neutrality. If we are genuinely committed to achieving educational equity, physical fitness must be recognized not as a luxury but as a fundamental right, essential to the academic, emotional, and physical well-being of all students.

## 5. Conclusions

In conclusion, this study revealed significant disparities in physical fitness outcomes among K–12 students in Connecticut following the COVID-19 pandemic. Students with disabilities, English Language Learners, and high-needs students had much lower rates of meeting the Healthy Fitness Zone compared to their peers. Racial and ethnic disparities were also evident, with Latin*, Black, and American Indian or Alaska Native students consistently performing worse than White and Asian students. At the district level, schools in affluent, predominantly White communities reported median fitness scores nearly four times higher than those in under-resourced, racially diverse districts.

These findings highlight systemic inequities in the allocation of educational and community resources, aligning with the principles of stratification economics, which emphasize that structural factors—not individual deficits—drive unequal outcomes. The slower recovery in physical fitness performance compared to academic subjects like ELA and math underscores how physical education remains underprioritized, especially for marginalized student populations.

To address these inequities, policymakers should integrate physical education into core educational frameworks, adopt equity-based funding models such as California’s Local Control Funding Formula, and ensure all states collect and report disaggregated physical fitness data. Additionally, culturally responsive physical education practices and inclusive programming should be implemented to ensure that every student, regardless of their background, has equitable opportunities for physical, cognitive, and emotional development. Without these systemic changes, disparities in physical health will continue to perpetuate broader patterns of educational and social inequality.

This study utilized publicly available, aggregated state-level datasets from Connecticut’s EdSight portal, which limited the analysis to descriptive statistics and broad trend identification. Due to the lack of individual-level data, it was not possible to conduct inferential statistical tests, perform multivariate analyses, or account for potential confounding variables such as school-level funding differences or geographic factors. Additionally, aggregated data may mask within-group differences and local contextual factors that could provide deeper insights into the observed disparities. While the findings highlight significant patterns and structural inequities, they should be considered indicative rather than definitive evidence of causal relationships. Future research using disaggregated, student-level longitudinal data would enable more precise modeling of causal pathways, more detailed subgroup analyses, and a clearer understanding of how intersecting factors such as race, language status, socioeconomic background, and disability influence physical fitness outcomes over time.

## Figures and Tables

**Table 1:** Five Connecticut school districts with the lowest Physical Fitness assessment scores, 2023–2024.

	Student Demographics	Jumoke Academy District	Goodwin University Educational Services (GUES)	Thompson School District	Torrington School District	Achievement First Bridgeport Academy District	Median Value
	Reaching Healthy Fitness Zone (%)	3.8%	15.8%	20.3%	20.4%	20.8%	20.3%
EL Status	English Learners	1.8%	13.5%	1.8%	14.8%	14.7%	13.5%
	Non-English Learners	98.2%	86.5%	98.2%	85.2%	85.3%	86.5%
Gender	Female	54.1%	50.7%	48.5%	46.9%	51.8%	50.7%
	Male	46.0%	49.3%	54.5%	52.9%	48.2%	49.3%
High-Needs	High-Needs	97.1%	72.9%	55.0%	75.9%	77.3%	75.9%
	Non High-Needs	2.9%	27.1%	45.0%	24.1%	22.7%	24.1%
Race/Ethnicity	Asian Americans	0.0%	2.6%	1.2%	4.1%	0.0%	1.2%
	Black Americans	94.8%	32.6%	0.9%	6.2%	52.7%	32.6%
	Latin*	4.3%	47.3%	7.8%	41.2%	43.4%	41.2%
	Two or More Races	1.0%	5.3%	3.1%	2.3%	1.4%	2.3%
	White	0.0%	12.3%	87.0%	45.9%	1.9%	12.3%
Special Education	Students with Disabilities	7.8%	17.6%	20.8%	19.2%	12.4%	17.6%
	Students without Disabilities	92.3%	82.5%	79.2%	80.8%	87.6%	82.5%

**Table 2:** Five Connecticut school districts with the highest Physical Fitness assessment scores, 2023–2024.

	Student Demographics	Capital Preparatory Harbor School District	Greenwich School District	New Canaan School District	Lisbon School District	Winchester School District	Median Value
Reaching Healthy Fitness Zone (%)		73.2%	74.1%	78.3%	80.2%	81.2%	78.3%
EL Status	English Learners	10.4%	5.5%	0.8%	1.6%	4.8%	4.8%
	Non-English Learners	89.6%	94.5%	99.2%	98.4%	95.2%	95.2%
Gender	Female	50.7%	47.2%	48.9%	47.5%	51.3%	48.9%
	Male	49.3%	52.8%	51.1%	52.5%	48.7%	51.1%
High-Needs	High-Needs	73.4%	32.4%	14.3%	50.5%	66.1%	50.5%
	Non High-Needs	26.6%	67.6%	85.7%	49.6%	33.9%	49.6%
Race/Ethnicity	Asian Americans	0.0%	0.0%	7.1%	0.0%	1.9%	0.0%
	Black Americans	66.3%	2.0%	1.3%	1.4%	2.1%	2.0%
	Latin*	33.7%	23.5%	6.9%	13.6%	15.1%	15.1%
	Two or More Races	0.0%	6.3%	5.4%	7.4%	3.6%	5.4%
	White	0.0%	59.4%	79.0%	77.7%	77.3%	77.3%
Special Education	Students with Disabilities	9.0%	15.4%	13.6%	21.3%	81.9%	15.4%
	Students without Disabilities	91.0%	84.6%	86.4%	78.7%	18.1%	84.6%

## Data Availability

The original data presented in the study are openly available in https://public-edsight.ct.gov/performance/physical-fitness-assessment?language=en_US (accessed on 25 June 2025).
